# Cyanobacterial Growth in Minimally Amended Anaerobic Digestion Effluent and Flue-Gas

**DOI:** 10.3390/microorganisms7100428

**Published:** 2019-10-09

**Authors:** Talita Beyl, Tobias M. Louw, Robert W. M. Pott

**Affiliations:** Department of Process Engineering, Stellenbosch University, Private Bag XI, MATIELAND 7602, South Africa

**Keywords:** anaerobic digester effluent, cyanobacterial cultivation, waste valorisation, nutrient recovery, biorefinery

## Abstract

Anaerobic digestion (AD) is an important industrial process, particularly in a biorefinery approach. The liquid effluent and carbon dioxide in the off-gas, can be used to produce high-value products through the cultivation of cyanobacteria. Growth on AD effluent is often limited due to substrate limitation or inhibitory compounds. This study demonstrates the successful cultivation of *Synechococcus* on minimally amended AD effluent, supplemented with MgSO_4_ and diluted with seawater. An 8 L airlift reactor illustrated growth in a pilot scale setup. Higher biomass yields were observed for cyanobacteria grown in diluted AD effluent compared to minimal medium, with 60% total nitrogen removal in the effluent. It was demonstrated that controlling the pH, increasing dissolved salt concentrations and adding MgSO_4_ to the effluent allowed for the successful cultivation of the cyanobacterium, circumventing the addition of clean water for effluent dilution. This could ultimately increase the feasibility of anaerobic digestion-microalgae integrated biorefineries.

## 1. Introduction

Sufficient nutrient supply is one of the major cost barriers of commodity chemical production from microalgae [1]. Generally, there are two approaches to improve process feasibility—lowering production costs or increasing product value. With the aim of improving nutrient utilisation and culture densities, progress has been made in terms of photobioreactor (PBR) designs [2,3,4] and commercialization of algal products has been worked towards for several decades now [5]. However, these studies often ignore or underplay the cost of nutrients necessary for continuous algal growth. Furthermore, if commodity chemical production by microalgal cultures is to be realised globally on non-arable land, a sustainable strategy with regards to water resource utilization must be developed. Not only must algae compete for water with the growing agricultural industry but microalgal growth also requires more water than most land crops, potentially triggering a "water versus fuel’ debate [6]. Subsequently, if the water and nutrient supply required for algae cultivation cannot be produced from existing low demand sources (such as effluent streams or sea water), the likelihood of industrial expansion is increasingly slim. This creates a need to investigate the integration of microalgae with alternative sources of water and nutrients.

Anaerobic digestion (AD) is increasingly being used to treat organic waste while producing biogas. AD effluent is typically characterised by a low chemical oxygen demand (COD) with high levels of residual nitrogen and phosphate [7]. Indeed, AD effluent often requires further treatment, since discharge into water bodies can lead to eutrophication while the high levels of ammonia are toxic to aquatic organisms. There is therefore both a need to develop methods for treating and reducing the impact of this increasingly significant wastewater and a potential demand for its components.

Bogan et al. [8] first suggested the intensive growth of algae in wastewater with the purpose of removing excess nutrients and algae have been identified as being particularly adept at nitrogen and phosphorous removal [9,10]. By combining AD with microalgal growth, the high costs of nutrient supply (nitrogen, phosphorous and water) can be significantly lowered. Carbon dioxide enriched air is also available from the AD process through the combustion of biogas, producing a CO_2_ enriched N_2_ flue-gas which can serve as a concentrated inorganic carbon source for the algae [11,12].

Research suggests nitrogen removal in excess of 90% can be achieved when microalgae are cultivated in (often diluted, treated or amended) AD effluents [9,13,14,15,16,17,18]. It is evident that a variety of algae species can fulfil this niche and that the technology can be applied to various anaerobically digested wastewaters [9,19]. Despite the apparent advantages, the use of AD effluent as microalgae growth media has found limited application due to, among other complications, high ammonia concentrations leading to toxicity [20]. Further, any process intent on utilizing AD effluent for microalgal growth will have to contend with the potentially reduced growth associated with light limiting turbidity. How exactly turbidity would affect such a process is a question still to be answered fully, since microalgae in general grow well under low light conditions [21] and so turbidity may not simply be a hindrance. Finally, the potential lack of all required mineral salts which cyanobacteria require for growth can be a limitation in these systems. Dilution has been used frequently in the literature to lower ammonia concentrations below the toxic threshold and decrease turbidity; dilution in these cases should ideally be performed with a carefully considered water source—utilizing scare freshwater resources defeats the end of reducing environmental impacts. Therefore the use of seawater for dilution is attractive [9,22].

Beyond wastewater remediation, cyanobacteria present opportunities for waste valorisation as well. The “techno-economic” feasibility of innovative waste-treatment bioprocesses is greatly enhanced if the potential to simultaneously generate high-value by-products is also present. Since cyanobacteria are amenable to genetic manipulation, they are promising candidates to produce a range of products, such as commodity or speciality chemicals [23]. Particularly in the context of a biorefinery, cyanobacteria may prove useful in nutrient recycling and value production [24]. Furthermore, *Synechococcus* strains are halophiles, suggesting the use of seawater as both a diluent (to decrease turbidity and ammonia toxicity) and a micronutrient source to be used in conjunction with AD effluents [25]. Seawater is inexpensive (abstraction being the major cost) and abundantly available (depending on location).

The aim of this study was therefore to assess the feasibility of the growth of *Synechococcus* PCC 7002 on minimally amended AD effluent diluted with seawater and to demonstrate the integration of AD with microalgal cultivation for wastewater treatment and value creation.

## 2. Materials and Methods

### 2.1. Growth Media Preparation

#### 2.1.1. AD Effluent

AD effluent was produced by combining cow manure and waste apples in a 50/50 weight ratio. The solid waste had a weight equal to 10% of the total working volume (100 g/1000 mL). Fresh, degassed anaerobic digestion inoculum was added as 10% of the final working volume (100 mL/1000 mL) and the mixture was made up with deionised water to the desired volume. CaCO_3_ was added to a final concentration of 5 g/L to buffer the solution and thereafter, 2 M HCl was added dropwise until the pH was between 6 and 6.5. The container was sealed and sparged with nitrogen for 10 min and placed in a water bath at 35 °C until all solid organic material was completely digested (approximately 21 days).

#### 2.1.2. AD Effluent Refinement

When experimentally required, the digestate was centrifuged in 200 mL batches at room temperature at a speed of 7607× *g* for 20 min to remove any sediment and most of the remaining solid particles. Thereafter, the centrate was vacuum filtered through 1 μm pore size glass fibre filters, 0.45 μm and 0.22 μm nitrocellulose respectively. At this point, the digestate was sterilised in an autoclave at 121 °C and 100 kPa for 15 min. All effluent used for experiments were refined in this way and all growth media (including synthetic media, AD effluent and synthetic seawater) sterilised as described. To minimise the loss of volatile fatty acids, the containers were completely closed during sterilisation, leaving enough head space in the container to allow pressure build up. Standard aseptic techniques were used to minimize contamination.

#### 2.1.3. Synthetic Media

D7 Synthetic growth medium was prepared as described in [26]. Macro nutrients were made up according to the recipe’s concentrations and certain nutrients that would precipitate in the solution were sterilised separately and added after the macro nutrient solution had been sterilised. Micronutrients (sterilised with the lid closed) were also added at this stage when all solutions have cooled down. Vitamin B12 was filter sterilised, using 0.22 μm syringe filters.

#### 2.1.4. Synthetic Seawater

Synthetic sea salt obtained from RED SEA^®^ was used to produce synthetic seawater to simulate the compounds present in natural seawater. The solution was made up by adding 38.2 g of synthetic sea salt to 1 L of deionised water whilst continuously stirring at high speed on a thermostatic magnetic stirrer. It was left to dissolve at 50 °C for 30 min and then sterilised. Approximate properties and concentration of nutrients are shown in Table 1.

#### 2.1.5. pH Control

The pH of the various media were adjusted by adding 1 M solutions of NaOH or HCl (autoclaved) while stirring under sterile conditions. A standard 50 mM phosphate buffer saline was prepared by adding 573 mg of Na_2_HPO_4_ and 583 mg of NaH_2_PO_4_ to each 500 mL batch volume for a final pH of 6.8. This was applied for modification of AD effluent in order to control the pH.

### 2.2. Experimental Framework and Growth Conditions

**Figure 1:**
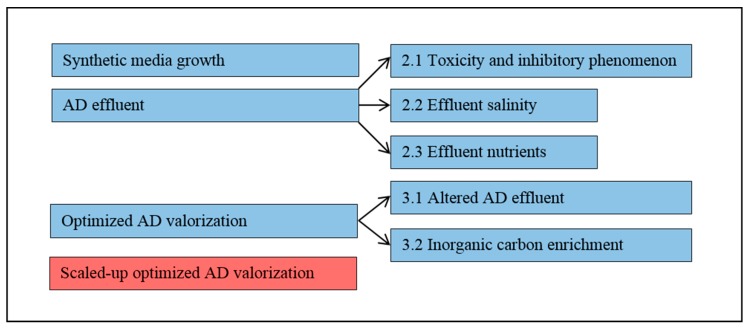
Schematic representation of the sequence of experimentation, showing flask-based experiments (blue boxes) and airlift reactor based experiments (red boxes).

The majority of experiments were conducted in an incubator where the conditions were controlled at a light intensity of 225 μE∙m^−2^∙s^−1^ (produced by neon bulbs), temperature of 38 °C, shaking speed of 150 rpm, media volume of 140 mL (in 250 mL baffled culture flasks) and algae inoculum of 10 mL. The variable parameters for each run were—the media, pH, headspace gas composition or a combination thereof. After flask experiments were concluded, the experiments were scaled up to an 8 L airlift reactor. Different aspects were investigated to evaluate growth in synthetic media and AD effluent. The experimental framework is shown in Figure 1. Conditions for each investigation shown in Figure 1 is detailed in Table 2. For investigation 1 to 6, batch mode experiments were performed in triplicates with a working volume of 150 mL.

#### 2.2.1. Growth on Synthetic Media Versus AD Effluent

Cultures were grown in i) synthetic medium (medium A D7), ii) pure AD effluent (prepared as detailed in Section 2.1.2) and iii) AD effluent with added micronutrients associated with medium A D7 to rule out nutrient limitation.

#### 2.2.2. pH Dependent Growth Experiments

To establish a pH operating window, nitrate (NaNO_3_) was replaced with ammonium sulphate ((NH_4_)_2_SO_4_) in a ratio that results in the same total nitrogen concentration (0.012 M). Seven sets of experiments were performed, with pH values ranging from 6 to 9 in increments of 0.5. The pH was adjusted back to the test condition using 1 M solutions of NaOH and HCl every 24 h.

#### 2.2.3. Ammonia Toxicity Experiment

For experimental set 3 listed in Table 2, the aim was to validate that ammonium was the preferred nitrogen source as opposed to nitrate and to examine the likely response of the organism to AD where both ammonia/ammonium and nitrates are present. Thus, the overall nitrogen concentration was kept constant at levels simulating synthetic media A D7 but the ratio of ammonium to nitrate was varied. Ammonia concentrations were varied from 0.1 M to 1.5 M at a constant light intensity, temperature and a pH of 6.7, in growth media under shake flask conditions (as detailed in Section 2.2).

#### 2.2.4. Growth in Amended AD Media

AD effluent was modified to address the limitations identified by elemental analysis by maintaining a pH of 6.8, adding MgSO_4_ in similar concentrations associated with the synthetic medium and diluting with seawater instead of synthetic media.

### 2.3. Airlift Reactor Growth Conditions

For investigation 7, the experiment was performed in an 8 L airlift reactor. It was designed as an internal loop concentric tube lift reactor built using acrylic as the construction material. The pneumatic closed system uses the velocity of sparged gas to circulate the fluid in cyclic patterns, as detailed in the initial work by Chisti and Moo-Yong [27]. Scale-up in this 8 L airlift reactor was demonstrated by growth in synthetic media and AD effluent. The culture was exposed to light produced by 2 fluorescent tubes giving a light intensity of 180 µE∙m^−2^∙s^−1^ and air sparged without CO_2_ enrichment at a flow rate of approximately 1 vvm. For the experiment in synthetic media, the average temperature was 23.4 ± 2.4 °C and for the AD effluent run it was 23.1 ± 3.2 °C.

### 2.4. Sampling

For the flask tests, all sampling and media handling was done under sterile conditions, to maintain monoculture within the shake flasks. Samples were taken five times a week, in volumes of 2 mL, for spectrophotometry. When nutrient elemental analysis was required, 1 mL extra was taken. Samples were also taken form the airlift reactor daily in volumes of 40 mL.

### 2.5. Biomass Quantification

Optical density (OD) of an axenic cyanobacteria culture was measured at 730 nm using a spectrophotometer (Spectroquant^®^ Prove 600: Powerful UV/VIS, Merck KGaA, Darmstadt, Germany). A calibration curve was generated to correlate OD to cell dry weight (CDW). For each triplicate sample to generate the standard curve, 10 mL of culture, of measured but varying OD_730nm_ was filtered using pre-weighed and dried 0.22 µm filter papers (Sigma), then rinsed with deionized water three times, dried at 60 °C overnight and weighed. A calibration curve relating OD_730nm_ to CDW was constructed, given as CDW (mg/L) = OD_730nm_/0.0022.

### 2.6. Nitrogen Quantification

Total nitrogen in growth media was measured to indicate nutrient depletion using Nitrogen Cell Tests (Merck KGaA, Darmstadt, Germany) 10–150 mg/L, in accordance with the manufacturer’s instructions. Briefly, 10 mL of sample is vacuum filtered using 0.22 μm filters to remove any cells and precipitate from the solution. For samples that fall outside the range of the test, dilutions were made using deionised water. Reagent 1 is added to the sample, mixed well and placed in a Spectroquant^®^ Thermoreactor TR320 (Merck KGaA, Darmstadt, Germany) at 120 °C for 60 min. Samples were allowed to cool down to room temperature without placing in a water bath. Reagent 2 was added to the reaction cell and well mixed for 1 min. 1.5 mL of cooled sample is pipetted into the reaction cell. 6 drops of reagent 3 was added, well mixed and allowed to react for 10 min. The reading was taken using the Spectroquant^®^ Prove 600: Powerful UV/VIS Spectrophotometer using the Total Nitrogen Test setting.

### 2.7. Turbidity Analysis

Turbidity was measured using a hand-held turbidity meter (Hanna Instruments HI 93703, Woonsocket, RI, USA).

### 2.8. Elemental Analysis

All other component concentration determinations were performed by the South-African Council for Scientific and Industrial Research analytical laboratories (Stellenbosch, South Africa).

## 3. Results

### 3.1. Substrate Limitations and Inhibition

#### 3.1.1. Comparison of Synthetic Medium and AD Effluent

**Figure 2:**
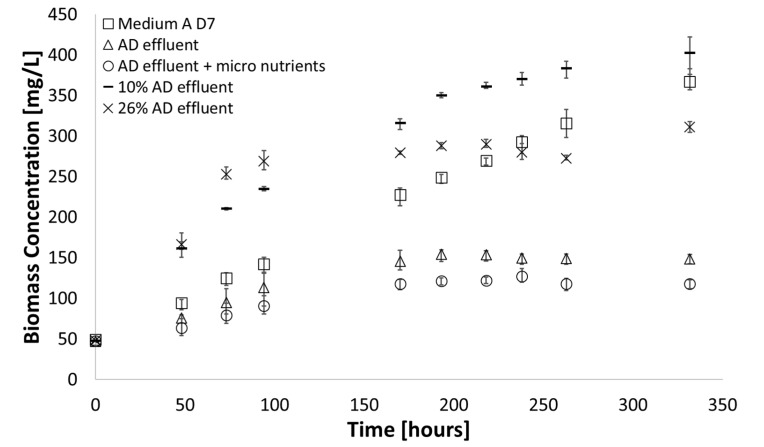
Growth observed in pure anaerobic digestion (AD) effluent, investigating micronutrient addition, diluted AD effluent at optimum dilutions as observed in literature and comparing with growth in medium A D7 (38 °C; 225 µE∙m^−2^∙s^−1^; pH = 8.2). Error bars represent the standard deviation of triplicate runs.

In order to evaluate the growth of the cyanobacterium in AD effluent a comparison between growth on minimal media, in which we expect to see stable growth and AD effluent was conducted and can be seen in Figure 2. The growth observed in pure AD effluent was limited but comparable to that in the synthetic medium during the first 100 h of the experiments. However, from approximately 150 h, the growth in AD effluent stagnated. The addition of micronutrients to the AD effluent had no positive effect on the growth observed, establishing that none of the micronutrients could be limiting the growth. All experimental conditions such as light intensity, temperature and mixing were fixed, suggesting nutrient limitation associated with a change in nutrient concentration within the AD effluent itself. It could be argued that ammonium assimilation, which causes a decrease in pH, reduced the pH below an acceptable limit, inhibiting the algae’s ability to grow. To support this argument, AD effluent was diluted with synthetic media to ensure all nutrients needed for growth are present, along with high salinities and raised pH values. The results are also shown in Figure 2.

The culture grown in 10% AD effluent accumulated 10% more biomass after 350 h compared to growth in pure medium A D7 and 170% more biomass compared to undiluted AD effluent. For the culture grown in 26% AD effluent, some growth was observed at approximately 100 h, like those grown in pure AD effluent, however, these cultures demonstrated only limited biomass increase and reached stationary phase at a low cell dry weight early in the growth curve. Thus, the limited growth in AD effluent could be circumvented to some extent through dilution. This supports the argument that a shift in pH caused growth to stagnate, as dilution with the synthetic medium would not only buffer the growth media but would also dilute the ammonia present in growth media, limiting potential toxic effects. Alternatively, it could be argued that a key nutrient was not present in the effluent in high enough concentrations and brought growth to a halt.

#### 3.1.2. Turbidity

The presence of dissolved and suspended material in anaerobic digestate can cause light attenuation and consequently potentially limit photosynthetic activity. To evaluate the effect of solids in suspension on turbidity, a series of clarified AD effluents, using first centrifugation and then filtration were prepared. This is clearly not a sustainable or suitable methodology for use in industrial application but nonetheless gives some insight into the effect of turbidity (which arises for the most part as a result of precipitated phosphates) effect.

Samples were taken of each processing step and tested for total phosphate concentration and turbidity, to confirm published findings [28]; the results obtained are shown in Table 3. The high turbidity associated with AD effluent is evident in the results obtained.

This analysis demonstrates that a significant portion of AD effluent turbidity arises from precipitated phosphate. This opens an opportunity for potential phosphate recovery as an additional step in this process. Without the specific aim of recovering a valuable product, it is certain that in industrial settings any clarification process, such as the one used here, would not be feasible. Thus, any process intent on utilizing AD effluent for micro-algal growth will have to contend with the reduced growth associated with light limiting turbidity.

#### 3.1.3. Ammonia Utilization and Inhibition

**Figure 3:**
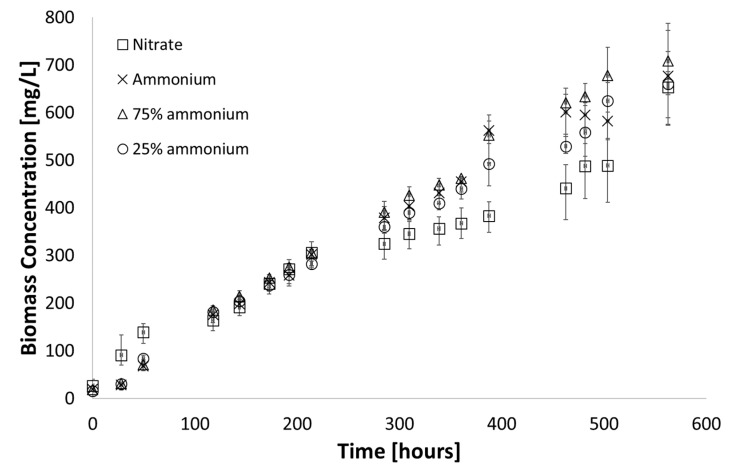
Growth observed when nitrate was replaced with ammonium as nitrogen source at the same concentration, (medium A D7; 38 °C; 225 µE∙m^−2^∙s^−1^; pH = 8.2). Error bars represent the standard deviation of triplicate runs.

**Figure 4:**
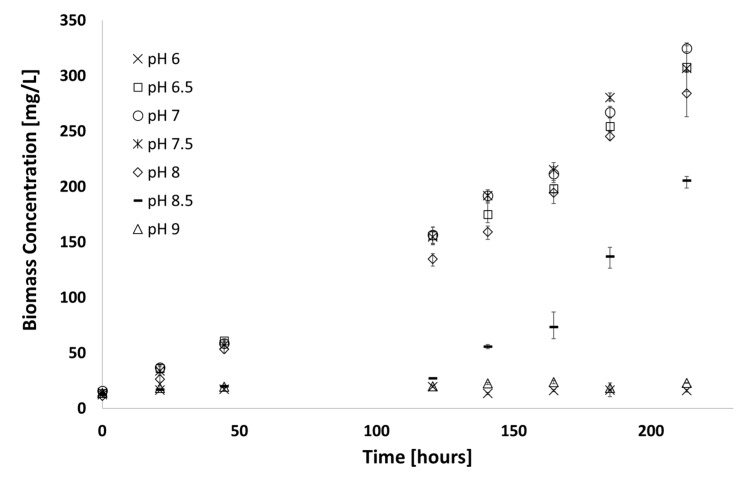
Growth observed in varied pH media A D7, (medium A D7; 38 °C; 225 µE∙m^−2^∙s^−1^; pH = 6–9). Error bars represent the standard deviation of triplicate runs.

By replacing the synthetic medium with AD effluent, the nitrogen source changed from nitrate to ammonia. Three sets of experiments were conducted to assess the cultures’ ability to utilize ammonia as a nitrogen source and the results are presented in Figure 3. All ammonium containing media showed better biomass yields than media containing nitrate as the sole nitrogen source, which confirms literature findings that these organisms preferentially utilize ammonium [29]. Indeed, ammonium is the preferred nitrogen source for these organisms not only because it requires less reducing power to incorporate ammonium into biomass than nitrate but cyanobacteria even repress nitrate assimilation in the presence of ammonium [30]. Since ammonium is in fact the preferred nitrogen source for these organisms, an opportunity exists to circumvent the toxicity issues of the high concentrations of ammonia present in AD effluent [20] by maintaining the culture at a lower pH, in which case chemical equilibrium favours speciation to ammonia. The results are shown in Figure 4.

With less than 10% variation in final biomass yield for cultures grown in media with a pH between 6.5 and 8, it was shown that the cyanobacteria could be grown in a more acidic environment (compared to a pH of 8.2 in the synthetic medium), enough to minimise the presence of molecular ammonia. However, growth was severely inhibited at pH ≤ 6.0 and pH ≥ 8.5.

**Figure 5:**
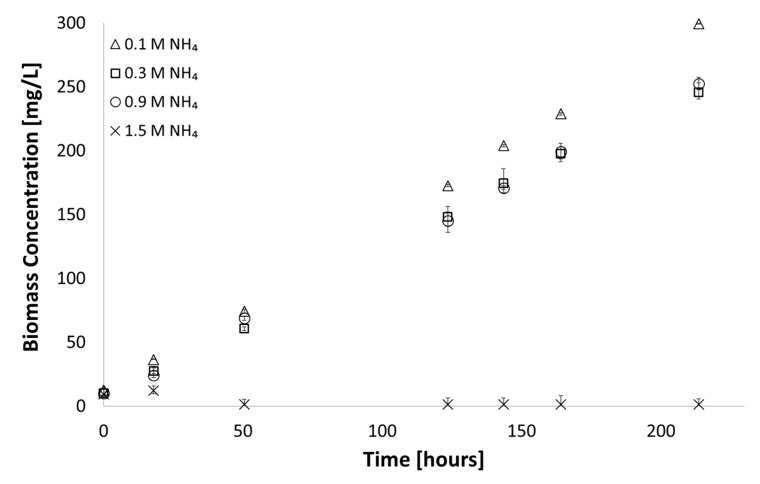
Growth observed in media with varying ammonia concentration at a constant pH (medium A D7; 38 °C; 225 µE∙ m^−2^∙s^−1^, pH = 6.8). Error bars represent the standard deviation of triplicate runs.

Through varying the added ammonium concentration, shown in Figure 5, it was clear that ammonium concentrations above 0.9 M affected cell growth negatively, even under pH sufficient to suppress speciation to the toxic ammonia.

It is clear that the pH limits to growth coincide with nutrient speciation and that excess ammonia inhibits growth even at a constant pH. Carbonate speciation shifts from bicarbonate to carbonic acid at a pH of 6.3 as the pH is lowered, thus it could be argued that the lowest operating pH is dictated by carbon speciation [19,31]. The upper growth limit of growth can be attributed to increasing ammonia inhibition, either due to an increase in total nitrogen or an increase in pH.

To test this hypothesis, a multiple substrate Monod growth model with inhibition was used to predict the scaled growth rate, µ$/{µ}_{max}$ as a function of the inorganic carbon (primary substrate), ammonium (co-substrate) and ammonia (inhibitor) concentrations, as shown in Equation (1):

(1) $$\frac{µ}{{µ}_{max}}\left(\frac{c_{IC}}{c_{IC}+K_{IC}}\right)\left(\frac{c_{NH_{4}}}{c_{NH_{4}}+K_{NH_{4}}}\right)\left(\frac{K_{NH_{3}}}{c_{NH_{3}}+K_{NH_{3}}}\right)$$

where c refers to the species concentration, K refers to the half-saturation constant and the subscripts IC, $NH_{4}$ and $NH_{3}$ refer to total inorganic carbon (that is, the sum of dissolved CO_2_, bicarbonate and carbonate species), ammonium and ammonia, respectively. The ammonium/ammonia speciation was determined using the ammonium equilibrium equation. The total inorganic carbon concentration was determined by assuming gas-liquid equilibrium. Note that the CO_2_ solubility increases with pH due to speciation into the bicarbonate/carbonate system. All physicochemical parameters were obtained at a temperature of 37 °C and a salinity of 35 g/kg [32].

**Figure 6:**
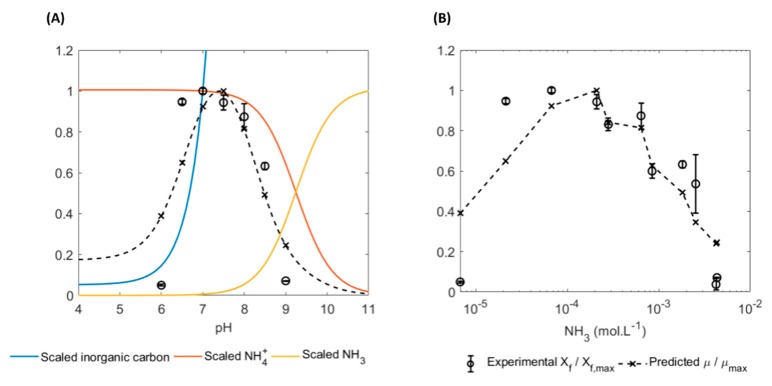
(**A**) Experimentally measured- and predicted scaled biomass growth with varying pH. The calculated total dissolved inorganic carbon, ammonium and ammonia concentrations are also shown. All variables are normalised to their maximum value, with the exception of inorganic carbon which is scaled to its value at pH of 7.0. (**B**) Experimentally measured maximum biomass concentrations (normalised to the maximum biomass concentration over all experiments), compared to the predicted scaled specific growth rate. Error bars represent the standard deviation of triplicate runs.

The predicted scaled growth rate µ$/{µ}_{max}$ was compared to the experimentally measured scaled final biomass concentration $X_{f}/X_{f,max}$, where $X_{f,max}$ is the maximum biomass concentration over all experiments (obtained at a pH of 7.0). Comparing µ$/{µ}_{max}$ to $X_{f}/X_{f,max}$ is appropriate as all the growth curves in this set of experiments were linear, such that $X_{f}\propto \mu$. The half-saturation constants $K_{IC}$, $K_{NH_{4}}$ and $K_{NH_{3}}$ (Equation (1)) were determined using non-linear least-squares regression, implemented using the MATLAB Optimization Toolbox (MathWorks, Nattick, MA). The results are shown in Figure 6.

Figure 6 shows that most of the pH dependent growth effects can be attributed to carbon- and ammonium speciation. The model also accurately predicts the effect of increasing total ammonium concentration at a constant pH. Model predictions were accurate with an average absolute error of less than 15%. 

The regressed values for the half-saturation constants were $K_{IC}$ = 5.67 × 10^−2^ mmol/L, $K_{NH_{4}}$ = 4.44 mmol/L and $K_{NH_{3}}$ = 1.19 mmol/L. Elemental analysis revealed that the ammonia levels in the AD effluent used (21 mmol/L; 15.35 mmol/L and 9.78 mmol/L) were higher than the $K_{NH_{4}}$ but ammonium concentrations would remain far below $K_{NH_{3}}$ as long as the pH was less than 9. The elemental analysis results are presented in Table 4, which details how each component in the AD effluent compares to standard media. The comparison allows for an estimation of likely limiting or inhibitory conditions, based on the organism’s sensitivity to each parameter. The results indicate that lack of growth in pure AD effluent was more likely caused by magnesium and/or sulphate deficiency, low salinity or high turbidity.

The results from the initial growth experiments in conjunction with elemental analysis reveals that AD effluent provides all the necessary macro- and micro-nutrients, with the exception of magnesium and sulphate. Furthermore, possible ammonia inhibition can be negated by maintaining a pH below 9.0. Finally, the low electrical conductivity and high turbidity may also limit growth.

### 3.2. Optimized Growth on Minimally Amended AD Effluent Diluted with Seawater

**Figure 7:**
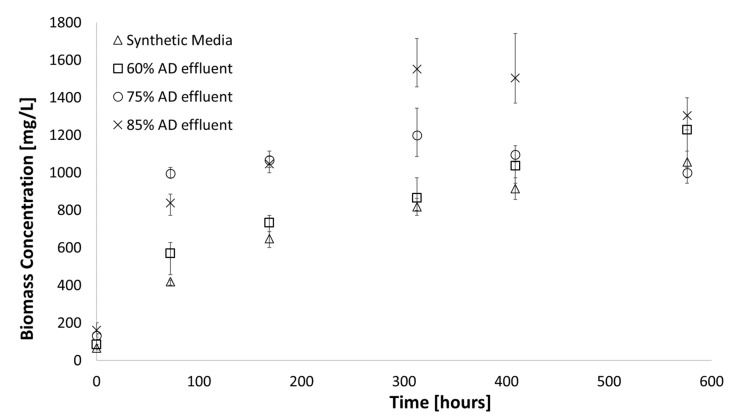
Growth observed in optimised AD media, adding MgSO_4_ to desired concentration and seawater in different levels (38 °C; 225 µE∙m^−2^∙s^−1^, pH = 6.8). Error bars represent the standard deviation of triplicate runs.

Observed growth (Figure 7) indicated that the study organism could be grown successfully in up to 85% AD effluent with a maximum biomass concentration of 1550 mg/L reached after 302 h for both 75% and 85% AD effluent, total nitrogen concentration remained constant after 312 h and thus no more ammonium utilisation took place – which should have shown a stationary phase in the growth curve.

**Figure 8:**
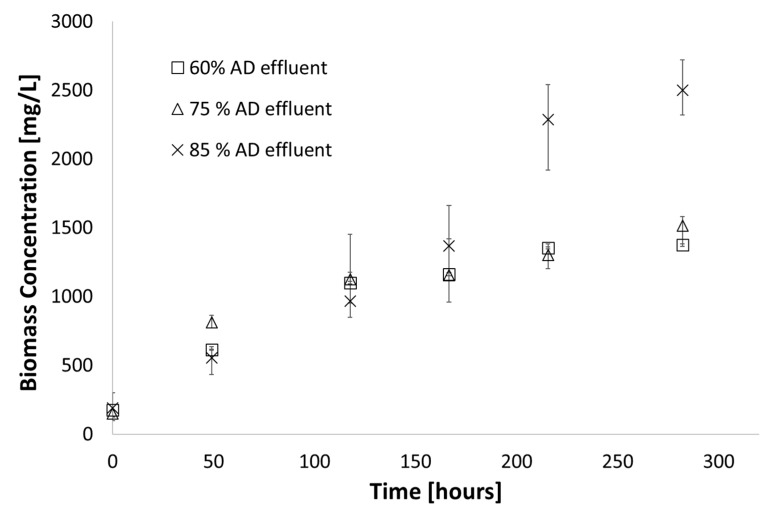
Growth observed in buffered optimised AD effluent (60%, 75% and 85%) with carbon enrichment (10% *v/v*) in the incubator head space gas (38 °C; 225 µE∙m^−2^∙s^−1^, pH = 6.8). Error bars represent the standard deviation of triplicate runs.

Experiments included carbon enrichment to simulate biogas addition from AD while buffering the growth media at a pH of 6.8. By doing this, the full potential of the resources AD can offer cyanobacterial growth are valorised. Although the transfer of inorganic carbon into the media will still be growth limiting, it is expected that the cyanobacteria will grow at a much faster rate, utilising more nutrients and ultimately depleting the nitrogen and phosphorous levels in the effluent. The results are presented in Figure 8. With the highest quantity of nutrients available to be utilised in 85% AD effluent, the potential limiting effect of carbon on cell growth was the most pronounced for this experiment. In 300 h, the addition of carbon in the headspace gas lead to a 38% increase in the biomass generated in 85% AD effluent.

### 3.3. Nitrogen Removal Efficiency

**Figure 9:**
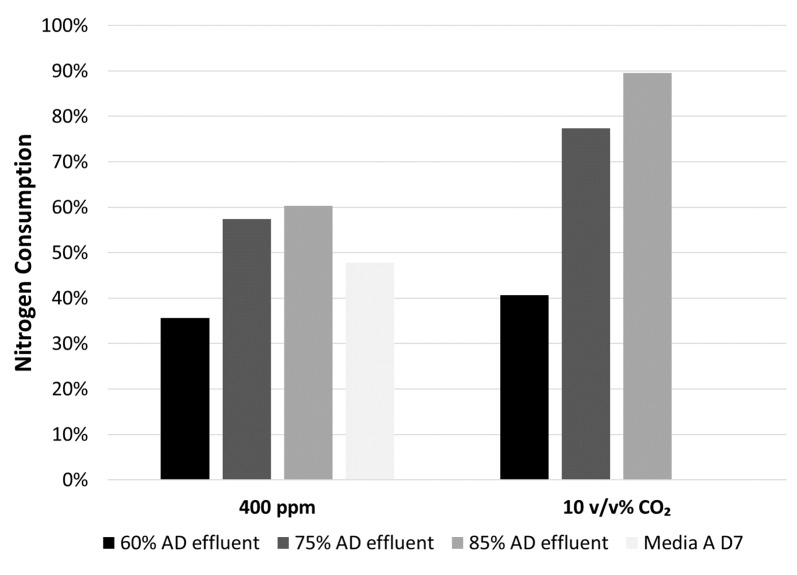
Nitrogen consumption observed for cells grown in buffered optimised AD effluent (60%, 75% and 85%) with carbon enrichment (10% *v/v*) in the incubator head space gas (38 °C; 225 µE∙m^−2^∙s^−1^, pH = 6.8).

Results regarding the consumption of nitrogen by microalgae is shown in Figure 9. The microalgae reduced the nitrogen content in 85% AD effluent down to 10% of its original concentration. With biomass yields significantly higher in the case of carbon enrichment for 60 and 75% AD effluent media, the depletion in growth rate could be due to another nutrient (such as phosphorous) being depleted. From these experiments, it was shown that the high concentration of nutrients present in AD effluent can be reduced up to 30% further by cyanobacteria when grown in a carbon enriched environment-supporting the hypothesis that the integration of AD effluent with microalgal growth is a means to lower the costs of large-scale algal production through waste valorisation.

### 3.4. Photobioreactor Scale-Up

**Figure 10:**
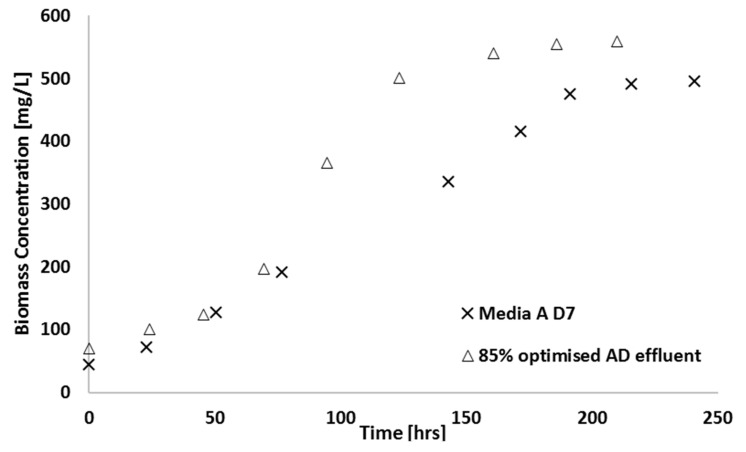
Biomass concentration observed for scaled-up growth in an 8 L airlift reactor using optimised AD effluent and sparged with atmospheric air (23 °C; 180 µE∙m^−2^∙s^−1^, pH = 6.8).

**Figure 11:**
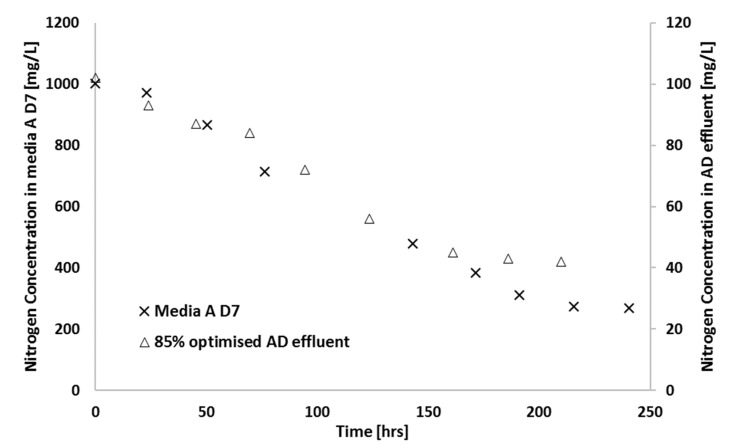
Nitrogen concentration observed for scaled-up growth in an 8 L airlift reactor using synthetic media and sparged with atmospheric air (23 °C; 180 µE∙m^−2^∙s^−1^, pH = 6.8).

As a demonstration of the scale-up of the shake flask work to bench scale, in order to understand which parameters become important in larger cultures, an 8 L airlift reactor was used to grow cultures on synthetic media on comparison to amended AD effluent. These types of experiments are particularly important in photosynthetic systems or those where mass transfer is important, since scale-up effects these parameters significantly. The results of these experiments are shown in Figure 10. The final biomass concentration achieved after 210 h was 560 mg/L and 500 mg/L in optimised AD effluent and synthetic media, respectively. For optimised AD effluent, nitrogen was reduced by 60% and the final biomass concentration achieved after 240 h was 500 mg/L. For synthetic media, nitrogen was reduced by 70% as shown in Figure 11.

The results indicate that the shake flask experiments provided an accurate representation of how larger scale cultures would behave – the amended AD effluent outperformed the synthetic media (likely as a result of more easily accessible nutrients and parallel phototrophic and autotrophic growth [33]). Importantly, the limitation of turbidity did not appear to affect the cultures significantly, although further experimentation may be needed to examine this complex phenomenon. Industrially, the integrated AD effluent, AD off-gas and algal cultivation has much promise and many researchers are investigating this around the world [34,35] but more work is required to fully understand the operating window required for these organisms and how best to control the systems.

Although these culture densities achieved are lower that seen in the batch reactors, it is important to note that these experiments were conducted with minimal operating condition optimisation to mimic the conditions under which the culture would be grown on an industrial scale – where the addition of heat and light would be unlikely, due to operational costs. The importance of the work lies in the fact that high concentrations of nutrients present in the effluent are significantly reduced whilst producing biomass, which in this case is a stand in for future valuable by-products.

It is envisioned that the use of minimally amended AD effluent diluted with seawater in coastal regions or saline ground water in arid, non-arable land will open new opportunities for microalgal cultivation. The use of halophilic or halotolerant microalgal species has the added advantage of providing additional resistance against contamination. *Synechococcus* is ideally suited to these conditions and its amenability to genetic manipulation can be leveraged in the production of high-value bioproducts without competing with food crops for land and water.

## 4. Conclusions

Between a pH of 6.5 and 7, the toxicity limit of ammonia was established to be between 0.9 M and 1.5 M. Without any carbon enrichment, cultures grown in seawater diluted AD effluent showed comparable growth to synthetic medium. With carbon enrichment (10% *v/v*), cultures grew 4 times faster. In the 8 L airlift reactor, higher biomass yields were observed when compared to growth in synthetic medium.

The work showed that *Synechococcus* can be grown to concentrated cultures in optimised AD effluent, diluted with freely available seawater. For this, the only inputs required are MgSO_4_ and pH control. This greatly reduces the nutrient costs and freshwater requirements associated with microalgal growth. The amenability of *Synechococcus* to genetic modification creates further opportunities for waste valorisation through the production of high-value by-products in a biorefinery context.

Currently, the major limitation to the proposed system is the low turbidity necessary to facilitate photosynthetic growth. The laboratory techniques used to clarify the AD effluent in this study are not suitable to large-scale implementation. Further research is required to identify industrially relevant clarification steps (such as flocculation and settling) and the potential effect thereof on microalgal growth.

## Figures and Tables

**Table 1:** Synthetic seawater properties.

Property	Value	Units
Specific gravity at 25 °C	1.0255	-
Salinity	35.5	g/kg
pH	8.2–8.4	-
Alkalinity	7.8–8.2	°dKH
Ca concentration	420–440	mg/L
Mg concentration	1250–1310	mg/L
K concentration	380–400	mg/L

**Table 2:** Experimental Conditions.

	Nutrients	pH	CO_2_ (GAS PHASE)
1.	Media A D7	8.2	400 ppm
2.	Undiluted AD effluent	8.2	400 ppm
	Undiluted AD effluent with added micronutrients (H_3_BO_3_; MnCl_2_.4H_2_O; ZnSO_4_.7H_2_O; Na_2_MoO_4_.2H_2_O; CuSO_4_.5H_2_O; NaVO_3_; CoCl_2_.6H_2_O)	8.2	400 ppm
	AD effluent diluted at 74% and 90% with deionised water	8.2	400 ppm
	Media A D7 adjusted as follows:		
3.	0.012 mol-N/L; 75% [(NH_4_)_2_SO_4_] + 25% [NaNO_3_]	8.2	400 ppm
	0.012 mol-N/L; 25% [(NH_4_)_2_SO_4_] + 75% [NaNO_3_]	8.2	400 ppm
	0.012 mol-N/L; 50% [(NH_4_)_2_SO_4_] + 50% [NaNO_3_]	6–9	400 ppm
	[(NH_4_)_2_SO_4_] = 0.1–1.5 M	6.5–7.0	400 ppm
4.	Synthetic seawater with added [NaNO_3_] = 0.012 M and [KH_2_PO_4_] = 0.37 mM	8.2	400 ppm
	Media A D7, with synthetic seawater replacing NaCl	8.2	400 ppm
5.	60%, 75% and 85% seawater diluted AD effluent and [MgSO_4_] = 0.2 M	6.8	400 ppm
6.	60%, 75% and 85% seawater diluted AD effluent and [MgSO_4_] = 0.2 M	6.8	10 *v/v*% CO_2_
7.	85% seawater diluted AD effluent and [MgSO_4_] = 0.2 M	6.8	400 ppm

**Table 3:** Clarifying step effectiveness and phosphorous correlation.

Sample	Turbidity (NTU)	*Turbidity* *(Classification)*	Total Phosphorous Reduction (%)
Raw AD	3525	Poor	0%
Centrate	362	Poor	15%
Filtrate (glass fibre)	253	Poor	20%
Filtrate (0.45 μm)	142	Fair	25%
Filtrate (0.22 μm)	6	Excellent	30%

**Table 4:** AD effluent and Media A D7 composition.

Property	Media A D7	AD Effluent	Conclusion
Potassium [mmol∙L^−1^]	11.70	14.91	Reasonable
Calcium [mmol∙L^−1^]	2.50	2.92	Reasonable
Magnesium [mmol∙L^−1^]	200.00	1.51	Possibly limiting
Total Nitrogen [mmol∙L^−1^]	12.00	17.08	Reasonable
Ammonia [mmol∙L^−1^]	0.00	15.52	Possibly inhibitory
Sulphate [mmol∙L^−1^]	200.00	0.19	Possibly limiting
Ortho-phosphate [mmol∙L^−1^]	0.36	11.83	Reasonable
Dissolved Organic Carbon [mmol∙L^−1^]	0.00	7.41	Reasonable
Total Organic Carbon [mmol∙L^−1^]	0.00	7.52	Reasonable
Electrical Conductivity [mS∙m^−1^ at 25 °C]	3500.00	291.00	Possibly limiting
Turbidity [NTU]	0.00	336.00	Possibly inhibitory
